# Case of Localized Tetanus

**Published:** 1918-09

**Authors:** 


**Case of Tetanus Localized in the Limbs.** By E. Chauvin, Médecin Aide-Major. *La Presse Médicale,* June 10, 1918.

The author reviews his conclusions drawn from six cases of localized tetanus that came under his personal observation. He rejects a limited conception of localized tetanus for one which would embrace all cases which begin with marked localization but later are generalized, or even fatal, and also those in which generalized symptoms appear first, but in which localization develops secondarily. In any case, there is a great variety of clinical pictures to be considered. The localized phenomena the author attributes to toxin conveyed through the nerves; the generalized phenomena, to that conveyed in the blood stream. The antitoxic serum, he believes, is capable of checking completely the spread of the toxin through the blood stream and hence it can prevent generalization if adequately administered at the right time. Sufficient serum administered so as to immunize the region of the wound may even prevent the impregnation of the nerves. Therefore, the author contends, the presence of localized tetanus means merely an insufficient serotherapy, and is not, as generally assumed, a modified form of infection. He urges the multiplication of preventive serum injections in all wounds that are slow in healing. If the localized tetanus has already appeared, indicating that the slow process of nerve absorption has set in, it may be maintained in the local stage by adequate serotherapy. Tonics and sedatives help in such cases to offset the strain of the spasm.

